# Self-assembling peptides for sciatic nerve regeneration: a review of conduit microenvironment modeling strategies in preclinical studies

**DOI:** 10.3389/fcell.2025.1637189

**Published:** 2025-08-13

**Authors:** Elena Stocco, Silvia Barbon, Annj Zamuner, Marta Confalonieri, Cesare Tiengo, Raffaele De Caro, Veronica Macchi, Monica Dettin, Andrea Porzionato

**Affiliations:** ^1^ Section of Human Anatomy, Department of Neuroscience, University of Padova, Padova, Italy; ^2^ Department of Women’s and Children’s Health, University of Padova, Padova, Italy; ^3^ Department of Surgery, Oncology and Gastroenterology, University of Padova, Padova, Italy; ^4^ Foundation for Biology and Regenerative Medicine, Tissue Engineering and Signaling, Onlus, Padova, Italy; ^5^ Department of Industrial Engineering, University of Padova, Padova, Italy; ^6^ Department of Civil, Architectural and Environmental Engineering, University of Padova, Padova, Italy; ^7^ Plastic Surgery Unit, Department of Neuroscience, University of Padova, Padova, Italy

**Keywords:** self-assembling peptides, luminal filler, ECM-like environment, regeneration, sciatic nerve, muscle

## Abstract

Effective nerve conduits development remains a significant challenge in regenerative medicine, with the potential to greatly improve patients’ quality of life in case of peripheral nerve injury. To date, several tubular devices have been introduced into clinical practice; however, the outcomes remain suboptimal. As empty conduits, lacking internal guidance structures or bioactive elements, they provide minimal support for nerve regeneration and fail especially in cases of long-gap nerve injuries. In this scenario, intense research efforts are directed toward improving conduit-associated results *in vivo*. Among the most promising strategies, the *in-situ* addition of luminal fillers has shown great potential in creating favorable microenvironment for axonal growth and tissue remodeling. Considering the many luminal fillers explored and reported in the literature, Self-Assembling Peptides (SAPs) have achieved significant attention by the scientific community due to their unique ability to arrange into biocompatible, extracellular matrix-like hydrogels that can favorably support axons and Schwann cells regeneration and organization within the conduit, guiding growth toward the distal stump. This review focuses on the use of SAP-based hydrogels as luminal fillers for sciatic nerve repair, summarizing the most relevant *in vivo* findings and highlighting their potential to enhance nerve regeneration.

## 1 Introduction

Peripheral nerve injury (PNI) is associated with substantial motor, sensory, and autonomic deficits descending from the loss of functions conveyed by the involved nerve, following axons continuity interruption, degeneration of nerve fibers distal to the lesion, and possible axotomized neurons death (Redolfi Riva et al., 2024). Currently, despite autograft is still broadly used as the “gold standard” clinical treatment option, nerve conduits are certainly considered a promising strategy to assist and support peripheral nerve regeneration, as acting as a guidance for axons growth (Shen et al., 2022). However, among available devices (eleven nerve conduits are approved by Food and Drug Administration, for clinical use) none adequately addresses PNI repair, guaranteeing satisfactory neuronal and muscular regeneration (Stocco et al., 2023a). This is true especially in cases of severe gap length; for nerve defects larger than 30 mm, there is no suitable evidence to suggest any comparable reconstructive option to the autograft (McMorrow et al., 2024). Within this scenario, intense efforts are dedicated towards the development of effective nerve conduits; together with good biocompatibility and biodegradation profile, nerve conduits are expected to promote the establishment of favorable microenvironment for nerve regeneration, resembling the native one (Shen et al., 2022).

In case of lesion, the peripheral nerve regenerative mechanism is characterized by the protrusion of growth cones from the proximal stump of the cut axons; the growth cones, sensing the surrounding environment can elongate whether they recognize the presence of a favorable substrate. Conversely, if the growth cones cannot reach the distal degenerating nerve, they twist and sprout within the proximal stump, giving rise to neuroma (Redolfi Riva et al., 2024). To date, to support adequate proximal/distal stumps re-conjunction, several approaches have been attempted providing for a more complex environment than that of common hollow conduits. Specifically, together with nerve conduits showing micro-groves and multichannels, also conduits intraluminal fillings have been explored: micro/nano fibers, spongy matrix, and hydrogels resembling the complexity of the extracellular matrix (ECM) may provide for guidance cues for nerve regeneration, showing certain mechanical properties to longitudinally instruct and support regenerating axons, as well as provide sufficient permeability for trophic support (Lopez-Silva et al., 2021; Yang et al., 2021a; Stocco et al., 2023a). Within this context, Self-Assembling Peptides (SAPs) hydrogels are included (Zhang et al., 2021).

SAPs consist of short peptide molecules self-assembling into stable secondary structures (α-helix, β-sheet, or random coil) and further forming various aggregation states (fibrils, fibril networks, membranes, and gels) under *in vivo* pH and ion concentration conditions. By adding physiological fluids to SAPs, the resulting hydrogels distinguish for a nanofibrous mesh, resembling the native ECM and undergoing natural biodegradation several weeks after implantation. Other characteristics include biocompatibility and non-cytotoxicity or immunogenicity *in vivo*. Interestingly, SAPs hydrogels can also safely encapsulate cells or drugs for specific therapies (Zhan et al., 2013; Tyler et al., 2013; Lu et al., 2018; Faroni et al., 2019). The SAPs most used as luminal fillers belong to the class of ionic-complementary peptides: sequences of 16 amino acids that alternate charged residues with hydrophobic residues and have a high propensity to assume a secondary β-sheet structure. Complementary ionic peptides can have a charge distribution that alternates a positive charge with a negative one (module I) or two positive charges with two negative charges (module II) or even four positive charges with four negative charges (module IV). The ionic-complementary SAP are usually indicated with three or four letters (amino acids included in the sequence), followed by the number of amino acids and the module (for example,: EAK 16-II or RADA 16-I).

This review focused exclusively on sciatic nerve repair, as it remains the most widely used injury model in preclinical studies. As reported in the literature, this preference is likely due to three key factors that include the relatively large size of the sciatic nerve (simplifying surgical manipulation) its easy anatomical accessibility and the need for results comparable with a substantial body of existing data, which mainly rely on this model (Geuna, 2015). The sciatic nerve injury model has been applied across a variety of animal species. Among them, rats are by far the most used (Zhou et al., 2025).

To the best of our knowledge, the effectiveness of SAPs as luminal fillers has not been evaluated in species other than rats or in strains other than Sprague-Dawley. As for the anatomical site, besides the sciatic nerve, only treatment of Sprague-Dawley rat’s recurrent laryngeal nerve (Yoshimatsu et al., 2020) was reported. Specifically, silicone tubes with/without RADA-16 were compared for the treatment of a 6 mm defect.

### 1.1 Peripheral nerve anatomy and extracellular matrix

The *endoneurium* (or endoneurial tube, endoneurial channel, or Henle’s sheath), is the innermost nerve layer. It directly surrounds the myelin sheath of each myelinated nerve fiber axon and clusters of small unmyelinated nerve fibers (Liu et al., 2018). It is characterized by a loose collageneous matrix, reticular fibers, nerve fibers, SCs, fibroblasts, endothelial cells, mastocytes and a thin network of capillaries and microvessels. This layer shows an outstanding intrinsic elasticity while providing only little mechanical protection to axons (Alvites et al., 2018; Zheng et al., 2023). Axons surrounded by endoneurium form a nervous fascicle (Gonzalez-Perez et al., 2013); in turn, each fascicle is wrapped by *perineurium*, individually. *Perineurium* is not a single layer tissue but a connective tissue of several concentric layers containing collagen fibers and flattened epithelium-like cells. Considering perineurium mechanical features, it is elastic and resistant to certain mechanical damage, thus supplying the nerve bundle for mechanical protection against tensile forces (Peltonen et al., 2013; Liu et al., 2018). Moreover, it also supports the blood-nerve barrier and nerve hemostasis, protecting the endoneurial environment in case of sudden changes of concentration in the vascular and extracellular spaces (Peltonen et al., 2013; Alvites et al., 2018). The *epineurium* is the outer coating layer of the nerve, mostly composed of collagenous ECM (Peltonen et al., 2013); it envelopes all fascicles and represents the 30%–70% of the nerve trunk in section. Specifically, its inner side directly coats all fascicles and their perineurium; blood vessels are recognizable here. Small amounts of adipose tissue can be also identified. The external epineurium side provides for nerve mechanical protection and anatomical shape (Alvites et al., 2018). While the endoneurium has a longitudinal orientation, the perineurium and the epineurium are circumferentially disposed. Epineural circulation consists of microvessels forming networks that extend longitudinally along the e*pineurium* and from which cross branches go through the *perineurium* to form a vascular plexus of capillaries at the level of the *endoneurium*. Peripheral nerve vascular system is extremely fragile; hence, morpho-structural modifications of the nerve can result in reductions in blood supply to residual levels. Considering the structural organization of the nerve, the collagen coatings assure for protection towards compression forces (Boissaud-Cooke et al., 2015).

Within the endoneurium, can be recognized both myelinated and unmyelinated axons, in close relation to the SCs. The axon itself distinguishes for a tubular shape with a cytoskeleton characterized by microtubules, microfilaments, and neurofilaments (Gallo, 2024; Ouyang et al., 2013). Microtubules are about 25 nm in diameter; they are straight, hollow cylinders consisting of a ring of 13 protofilaments that are made of alpha and beta tubulin heterodimers (Ouyang et al., 2013; Sánchez-Huertas and Herrera, 2021). Microfilaments are about 5 nm in diameter; they are identifiable throughout the neuron, mainly in the neuritis. They are made of two thin strands which are actin polymers, constantly undergoing assembly/disassembly. Neurofilaments are about 10 nm in diameter. They have multiple subunits organized in a chain-like structure and each subunit has three protein strands woven together; in each strand is recognizable a long chain of protein molecules that are coiled in a tight and springlike configuration. The cytoskeleton supports the maintenance of axons morphology, polarity and integrity while participating in axonal growth, promoting proteins and organelles transport between the cell body and the axon terminals (Ouyang et al., 2013; Kevenaar and Hoogenraad, 2015).

Schwann cells (SCs) are glial cells divided into two phenotypes: myelinating SCs and non-myelinating SCs. In myelinated axons, each SC is associated, one-to-one, with a single axon and it produces several layers of cell membranes forming the myelin sheaths (Chen and Strickland, 2003). The space between two myelin segments (or internodes) is occupied by demyelinated spaces (or nodes of Ranvier). Myelin, insulating the axons electrically, specifically increases the rate at which neural electrical impulses propagate along the nerve fiber (saltatory propagation). Conversely, unmyelinated SCs surround multiple small diameter axons without producing myelin (Nocera and Jacob, 2020). The saltatory propagation of action potentials along the axon has fundamental role in establishing functional connections with the terminal organs (i.e., muscle fibers or sensorial terminations). Schwann cells are responsible for production of ECM, neurotrophic factors, cell adhesion molecules, and other molecules supporting nerve regeneration and providing a favorable environment for cells in tissue (Rasband and Peles, 2015).

In the peripheral nerve, the ECM is a three-dimensional (3D) network found in the endoneurium and basal lamina, providing a suitable niche for the SCs growth (Gonzalez-Perez et al., 2013; Xiao et al., 2016). It is made up of molecules guaranteeing for cells structural support, favoring their alignment and creating a physical pathway for their movement/extension (Chen et al., 2015; Xu et al., 2020; Yu et al., 2023); moreover, interacting with different growth factors, signal receptors, and adhesion molecules, the peripheral nerve ECM influences cellular behavior and function and is also essential for proper myelin formation around axons (Ning et al., 2019; Yu et al., 2023): ECM and SCs are in deep interaction in both physiological and pathological conditions (Yu et al., 2023; Gao et al., 2013). Collagen type IV and the glycoproteins laminin and fibronectin can be mainly recognized in the ECM of the peripheral nerve (Gao et al., 2013; Yang et al., 2023).

Collagen type IV is a principal structural component of the basal lamina; together with its receptors, it promotes the adhesion/migration/myelination of SCs as well as neurite growth (Chernousov et al., 2008; Yu et al., 2023). Laminin regulates SCs proliferation and survival and it is essential for axons myelination. Without laminin, SCs can migrate along the axons, populate the peripheral nerve, and proliferate normally, but cannot differentiate into a myelinating phenotype (Chernousov et al., 2008; Xiao et al., 2016). Laminin acts as an interesting nerve regeneration-stimulating factor; in particular, two short laminin peptides (YIGSR and IKVAV) have been shown to specifically bind to the surface targets of neurite processes and regulate axon adhesion/migration. Through the modulation of filopodia and lamellipodia dynamics, at the tip of the growth cone, laminin inhibits growth cone retraction but also promotes axon growth and directional extension (Ji et al., 2020). Fibronectin can promote SCs growth and motility (Yu et al., 2023); it is also present in the endoneurium, in the areas surrounding individual axon-Schwann units (Chernousov and Carey, 2000). Fibronectin-laminin combination enhances peripheral nerve regeneration across long gaps (Bailey et al., 1993).

Specific ECM molecules do not act as individual units, but as part of a highly organized macromolecular complex; therefore, the assembled ECM displays biochemical and physical properties that are certainly not showed by each ECM molecules alone (Chernousov and Carey, 2000). The interaction between SCs and the ECM is crucial for the remyelination process (Yu et al., 2023).

### 1.2 Clinical classification of peripheral nerve lesions and regeneration mechanism within conduits

According to damage severity, three different types of PNI were identified by Sunderland in 1951: neurapraxia, axonotmesis, and neurotmesis. Neurapraxia and axonotmesis descend from compression, overstretch, and nerve crush; the related symptoms are moderate, and prognosis is certainly better than that associated with neurotmesis. In fact, the basal lamina around axons/SCs and the connective neuronal stroma (endoneurium, perineurium, and epineurium) is preserved, leaving an intact and permissive environment for axonal regeneration. Conversely, neurotmesis (nerve transection) distinguishes for nerve continuity disruption, associated with partial or complete alteration of the connective tissue. Hence, here, no physical guidance and biochemical support (originally provided by basal lamina tubes and connective tissues) is still identifiable between the proximal and distal stumps of the injured nerve, with consequent regenerating axons misorientation (Rao et al., 2022). In case of neurotmesis the main aim of PNI treatments is to bridge the nerve stumps, guiding severed axons to reach their disconnected target.

Peripheral nerve regeneration within a nerve conduit starts with the formation of a fibrin cable, bridging the gap between the proximal and the distal stumps. This structure acts as a guiding surface, thus favoring the ingrowth of fibroblasts, blood vessels, and SCs migrating from both stumps. Progressively, it is replaced by collagen (deposited by the invading fibroblasts) and laminin (produced by the SCs) organized in fibrils with a longitudinal orientation; moreover, the conduit lumen fluid is enriched by trophic factors. Then regenerating axons can grow from the proximal stump along the newly formed natural scaffold. Failure of the initial cable formation or limited supply of migrating cells into the tube, due to excessive length of the gap, lead to failure of regeneration (Gonzalez-Perez et al., 2013; de Luca et al., 2014). Within this scenario, prefilling nerve conduits with ECM proteins (including collagen, laminin, hyaluronic acid and fibronectin) or with artificial scaffolds can enhance the regenerating environment with a positive impact over nerve regeneration (Behtaj et al., 2022).

### 1.3 SAPs as nerve conduits luminal filler

SAPs stood out in the field of nanotechnology and biotechnology for possible application in tissue engineering, including nerve regeneration (Yang et al., 2020; Sun et al., 2016). Artificial nerve conduits are expected to support the reconjunction of the proximal and distal stumps also creating a regenerative environment allowing for axons sprouting while reducing the formation of scar tissue. To achive this, conduits should replicate the complexity of native nerve characterized by endoneurial channels, tissue-specific ECM and growth factors, and vascular components. Modifications of the inner lumen of the artificial conduits with physical cues that could potentially guide the regenerative process in a confined environment is highly appealing (de Luca et al., 2014; Perrelle et al., 2013).

Self-assembly is a spontaneous and reversible process where disordered components can form hierarchically organized structures through specific non-covalent intermolecular interactions, including hydrogen bonds (also that mediated by water), van der Waals forces, ionic, electrostatic and hydrophobic interactions. Despite being weak whether considered alone, a certain strength derives in case they are together, improving supramolecular structures stability (Koutsopoulos, 2016; Whitesides and Grzybowski, 2002; Wang et al., 2016; Li et al., 2021).

Many peptides have been used to develop 3D biomaterial scaffolds; typically, they are divided into three groups, namely, α-helical peptides, β-sheet peptides and collagen-mimetic peptides. Among them, β-sheet peptides are the most widely used due to their ability to support peripheral nerve regeneration by promoting cell migration, slow nutrients/growth factors absorption and metabolic waste depletion (He et al., 2014; Huang et al., 2021). Moreover, SAPs modification through functional motifs, including controlled release of bioactive signals, can increase similarities between SAPs to native ECM, in turn improving interactions with cells (He et al., 2014).

Although the design of SAPs is based on well-known principles, the approach to that remains largely empirical. There are three common methods for the development of SAPs: i) empirical design: this strategy develops SAPs that are inspired by those naturally present in living systems and it is based on trial-and-error methods; ii) computational screening: this strategy uses computer modeling to predict short peptides behaviour in water; iii) dynamic peptide libraries: this strategy uses enzymatic reactions to develop peptide sequences, enabling discovery of self-assembling candidates (Yang et al., 2021a).

The structural characterization of SAPs is fundamental for understanding their properties and predict effectiveness whether adopted in biomedical field and for regenerative purposes. Following characterization, it is possible to ameliorate SAPs design and the associated functions (Alvarez-Fernández et al., 2025).

#### 1.3.1 SAPs preparation, characterization, and importance of structural properties over nerve regeneration

##### 1.3.1.1 Synthesis and functionalization effects on assembly

Self-assembling peptides used as fillers are usually produced by solid-phase or solution-based peptide synthesis. When self-assembling sequences are intended to be conjugated to bioactive sequences (i.e., peptides/proteins capable of modifying cellular behavior like adhesion, proliferation or differentiation), two strategies may be employed: i) synthesis of the entire biomolecule (self-assembling sequence + bioactive sequence) in a single process or ii) conjugation of the two sequences by chemoselective ligation recurring to bio-orthogonal functional groups that can react specifically and under mild conditions to form the final conjugate. Hence, following chemical synthesis, chromatographic purification ensures a high degree of homogeneity in both SAPs and their conjugates.

Functionalization of SAP hydrogels with varying concentrations of bioactive sequences, achieved by mixing different ratios of conjugates with plain SAPs, allows identification of the most biologically effective derivatives. Interestingly, the presence of conjugates within the SAP matrix does not inhibit the hydrogel formation but it influences fiber morphology, typically resulting in increased fiber diameter while preserving β-sheet-driven nanofiber formation.

##### 1.3.1.2 Structural characterization and rheological evaluation of SAP hydrogels

Considering that morpho-structural and mechanical characteristics of the luminal filler can influence the biological interactions between the filler and the regenerating nerve tissue, with impact over nerve repair, their characterization is crucial. To this purpose, different techniques can be adopted including Atomic Force Microscopy (AFM), to observe the nanofibers formation and their 3D organization; Scanning Electron Microscopy (SEM), for ultrastructure; Circular Dicroism (CD), to analyze the secondary structure; and vibrational spectroscopy (including FT-IR and Raman) for detailed information on molecular interactions, secondary structure, and the self-assembly process. Lu et al. (2018) showed that the inclusion of conjugates at a 1:1 ratio in the RADA peptide does not affect the length of the fibers but modifies their diameter. Similarly, Yang et al. (2020) proved that conjugation with longer bioactive motifs further increases fiber thickness, from approximately 15 nm in plain RADA to ∼18 nm in RADA/IKV and ∼30 nm in RADA/IKV-GG-RGI, (1:1 ratio). Brun et al. (2019) further demonstrated the role of sequence variation in nanofiber organization (and morphology): sequence modifications significantly influence nanofiber organization, with EAK forming densely packed, aligned fibrils, whereas the EAbuK variant (Ala→Abu or α-aminobutyric acid) produced fewer and shorter fibers; RGD or IKVAV functionalization led to layered structures with compact or aggregated fibrils, highlighting the role of both primary sequence and bioactive modification in critically modulating SAP architecture.

Self-aggregation mechanism for ionic-complementary peptides relies on their ability to adopt a β-sheet conformation. Since the sequences are composed of alternating charged polar and non-polar residues, the beta-sheet structure is characterized by a spatial arrangement in which all the non-polar chains align on one face of the beta sheet while all the charged chains (both positive and negative) are on the opposite face of the β-sheet. It is the coupling of the non-polar faces that produces the formation of fibers. For this reason, techniques that study the secondary structure and the conformation of SAPs and their conjugates are important for understanding and controlling their self-assembly properties.

To evaluate whether the introduction of bioactive motifs affects peptide secondary structure, far-UV CD is commonly used. Several studies have examined the impact of conjugation on β-sheet formation: Lu et al. (2018) showed that RADA mixed (1:1) with neurotrophic motifs (from Nerve Growth Factor, NGF or Brain-derived neurotrophic factor, BDNF), exhibit a lower β-sheet content, suggesting that the sequences may interfere with proper self-assembly. Similarly, Yang et al. (2020) confirmed that conjugates of RADA with motifs like IKVAV or RGI adopt random coil conformations, while mixtures with unmodified RADA restore the β-sheet signal, suggesting that co-assembly can maintain structural order. However, increasing the size of the attached motif reduces β-sheet intensity, reflecting a balance between biological functionality and structural integrity. In contrast, Shen et al. (2022) reported that RADA-based hydrogels covalently conjugated with KLT from VEGF and IKVAV from Laminin, preserved their β-sheet signature, even in complex multi-conjugate formulations. Likewise, Brun et al. (2019) reported that EAK-based conjugates maintained a β-sheet conformation, even with large or dual bioactive domains, suggesting that this SAP may tolerate functionalization better than RADA. Overall, these findings highlight that maintaining β-sheet content is critical for ensuring the structural and mechanical functionality of SAP hydrogels. In fact, despite bioactive conjugation is fundamental for biological performance tuning, an excessive disruption of β-sheet structure can impair fiber formation, and in turn compromise hydrogel integrity, reducing their use as scaffolds or injectable fillers in regenerative medicine. Therefore, a careful balance must be achieved between introducing functionality and preserving the self-assembling architecture required for material performance.

Vibrational spectroscopy has been successfully used for the structural characterization of SAPs with the amide I band (1,695–1,630 cm^-1^) being especially informative for identifying β-sheet conformations, essential for hydrogel formation. Di Foggia et al. (2011) demonstrated how vibrational analysis can track conformational changes and intermolecular interactions in SAPs under varying physical conditions. More functionally, FT-IR has been used to assess the structural integrity of peptide conjugates: Pugliese et al. (2022) confirmed β-sheet retention in RADA conjugates with plant-derived bioactive peptides, while Fujii et al. (2021) showed that the site of motif conjugation (N- vs C-terminal) influences the β-sheet vs β-turn balance—an important factor affecting fiber assembly and hydrogel performance.

The secondary structure of SAPs influences the size of the fibers and ultimately affects the rheological properties of the hydrogels, that are both critical factors for their suitability in biological applications. Key rheological parameters include the storage modulus (G′), reflecting stiffness; loss modulus (G″), related to viscous behavior; and the loss factor (tan δ = G′′/G′), describing the balance between elasticity and viscosity. These parameters together define the hydrogels’ viscoelastic behavior (Yan and Pochan, 2010; Fu et al., 2021). SAPs rheological properties can be influenced by several factors including peptide concentration, pH value, ionic strength and shear-thinning properties. From peptide concentration descends nanofibers density and entanglement: stiffness and robust networks increase along with it; pH values affect electrostatic interactions between peptide chains, thereby having a role over nanofibers aggregation and network compactness; ionic strength influences gelation dynamics and stiffness altering the peptide-related charge (Fu et al., 2021; Pang et al., 2024).

In the study by Lu et al. (2018), no significant variation was observed between the hydrogels containing only RADA and those enriched with 50% conjugates; in both cases, the Young’s modulus was around 1.39 ± 0.19 KPa, closely matching the range of values typical of nervous tissue (0.5–3 kPa). Contextually, Yang et al. (2020) reported hydrogels with G′ around 3000 Pa, showing elastic properties similar to nerve matrix. Particularly, peptide concentration remains fundamental in determining its mechanical performance. A higher peptide concentration in the gel results in a higher G′ value, as evidenced by studies showing up to 10-fold increases in G′ upon doubling or quadrupling peptide levels (Koch et al., 2018; Clarke et al., 2018; Sieminski et al., 2007; Schneider et al., 2002). This correlation can be attributed to the fact that in higher concentration hydrogels more fibers form, leading to additional entanglement and cross-linking into a firmer network.

Peptide sequence modifications also impact rheology: substituting valine with less hydrophobic residues like alanine or serine reduces gel stiffness and resilience (Ramachandran et al., 2005). Warren et al. (2023) observed that altering the terminal amino acid from glutamine to serine, the viscoelastic properties of the hydrogel varied. Specifically, G′ was reduced by two to three orders of magnitude likely due to reduced peptide cohesion.

The SAPs rheology is affected, not only by intrinsic characteristics of the material, but also by the external physico-chemical conditions, that can influence the peptide secondary structure. Hence, Schneider et al. (2002) demonstrated that the gelation is reversible by changing pH.

As previously described, a nerve conduit luminal filler should provide physical support to guide axonal regeneration across the lesion and create a microenvironment ideal for growth, migration and differentiation of cells involved (Ryan et al., 2017; Wu et al., 2018; Kohn-Polster et al., 2017).

##### 1.3.1.3 Design criteria for SAP-based injectable hydrogels

In the design of an injectable hydrogel filler, the mechanical specifications, like mechanical stiffness and shear-thinning capability, have to be balanced with biological activity that the filler should elicit (Kohn-Polster et al., 2017). Thus, the main mechanical requirement for the biomaterials employed for filler fabrication is having an adequate stiffness, that could trigger mechano-transduction phenomena on neuronal stem cells (Kohn-Polster et al., 2017). Within this context, SAPs emerge as compelling materials due to their easily tunable stiffness, varying, for example, the peptide concentration. The effect of SAPs stiffness on cell behavior was investigated by Sieminski et al. (2007); human umbilical vein endothelial cells (HUVECs) on RAD16-I and RAD16-II gels with different stiffness, ranging from 40 to 735 Pa showed specific morphology, 3D disposition and interconnections, highlighting a more adequate behavior on softer supports (Sieminski et al., 2007). Regarding peripheral nervous tissue, the optimal bulk stiffness was identified as 1 kPa (Ryan et al., 2017; Wu et al., 2018; Kohn-Polster et al., 2017).

A further requirement for intraluminal nerve conduit filler is related to its positioning process; in fact, in case of injectable fillers, the material should have appropriate self-healing properties (Ramachandran et al., 2005; Schneider et al., 2002; Kohn-Polster et al., 2017). Specifically, during the injection the strain exerted on the gel should disrupt any physical cross-links, decreasing considerably the material viscosity. Once *in situ*, the hydrogel should be able to quickly recover, reforming after cessation of shear due to the quick relaxation time of the molecular self-assembly process (Clarke et al., 2018). Ramachandran et al. tested how SAPs-based hydrogels responded to shear-induced breakdown (Ramachandran et al., 2005). Two different decapeptides (i.e., Acetyl-WK(VK)4-amide and Acetyl-EW (EV)4-amide) underwent single and multiple cycles of break-and-recovery (break: continuous 200% sine-wave strain for 2 min, recovery: constant 0.2% strain at 1 rad/s frequency for 30 min). In the recovery process, two phases were observed: (i) an initial phase lasting few seconds characterized by the obtaining of about. 50% of the gel mechanical strength, indicating a fast recovery of the nanofibrillar network; (ii) a slower phase in which the material re-gains the remaining mechanical strength within a few hours. Thus, this study proved the dynamic nature of SAP gels, in which β-sheets can gradually assume more energetically favorable and mechanically robust conformations, making the shear-induced breakdowns completely or almost completely reversible.

Lastly, the resilience of SAPs hydrogels was extensively investigated, revealing these gels are incapable of withstanding strains exceeding 10% (Ramachandran et al., 2005; Koch et al., 2018; Clarke et al., 2018; Goktas et al., 2015). For example, decapeptides developed by Ramachandran et al. (2005) presented yield strains lower than 2%, whereas Goktas et al. (2015) measured a yield strain of a self-assembled peptide amphiphile below 0.5%. Beyond the yield strain, the hydrogel starts to break, and the material displays fluid-like behavior (i.e., shear-thinning). In addition, Koch et al. (2018) found a correlation between peptide concentration and material resilience. Thus, it was observed a decrease in the strain tolerance with the increase of concentration.

Overall, a SAP can be considered an effective luminal filler for nerve regeneration when it combines specific structural/mechanical/biological properties. As for structure, it must form stable β-sheet-driven nanofibers, mimic the ECM, and thus provide a scaffold that supports axonal growth. Regarding the mechanical behavior, it is expected to show tunable stiffness (about 1 kPa to match native nerve tissue), and possess shear-thinning and self-healing capabilities, thus allowing for easy injection and rapid recovery of structure *in situ*. Considering the rheological behavior, the SAP should balance elasticity and viscosity as the derived hydrogel needs to be both soft and stable. 1t should also recover its own structure following injection and respond well to conditions (i.e., pH) changes. SAPs must also support the incorporation of bioactive motifs (e.g., IKVAV, RGD) without compromising fiber architecture, enabling functionalization that enhances cell adhesion/migration/differentiation. The ideal SAP luminal filler must integrate mechanical support with biological functionality to create a permissive environment, ECM-like, for nerve regeneration.

## 2 Methodology

### 2.1 Literature search strategy

A systematic review was performed in accordance with the Preferred Reporting Items for Systematic Reviews and Meta-Analysis (PRISMA) recommendations. Two investigators (E.S. and S.B.) independently searched PubMed and Scopus databases to find articles published on this review topic. The following terms were used: [(“self assembling peptide” AND “peripheral nerve” AND “regeneration”)]. Our search was limited to studies published in English and was updated until 25 February 2025.

Upon removal of duplicates, each study was considered by two researchers, independently. Preliminarily, abstracts and methods were considered to verify pertinence. Finally, included studies were reviewed.

### 2.2 Inclusion and exclusion criteria

Studies published in English up to 25 February 2025 were eligible for inclusion in the present study; no temporal limitations defined the starting point of the literature search. Abstract availability was required for initial screening. Studies were required to present data from *in-vivo* studies using natural/synthetic conduits filled with SAPs as luminal fillers for the repair of sciatic nerve lesions with substance loss.

Studies published in languages other than English were excluded from the present study. Studies with no available full-text were excluded. Review articles, opinion articles, book chapters, conference paper, and studies based solely on *in vitro* data were excluded. Studies investigating lesions to different anatomical regions/nerves than the sciatic nerve were excluded. Studies in which SAPs were incorporated into the conduit wall rather than used as a luminal filler were excluded.

## 3 Results

### 3.1 Study selection

**FIGURE 1:**
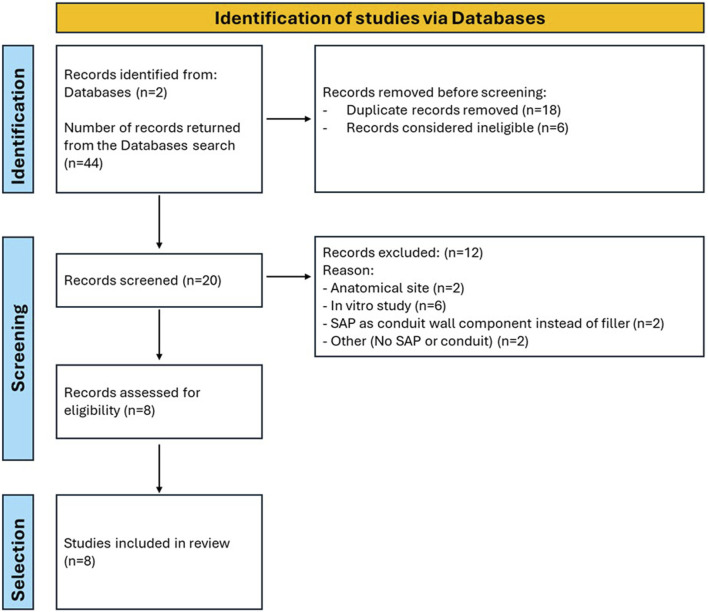
Flowchart of the search process.

A preliminary query of the PubMed and Scopus databases revealed 17 and 27 studies from 2007 to 2025 (Figure 1). After an initial screening using the titles, the abstracts and methods, eight studies were found to meet our inclusion criteria. These eight studies in which SAPs were used as luminal fillers are reviewed in Supplementary Table 1. Studies on SAPs as luminal filler spanned from 2013 to 2022. Two studies focused on the SAP RADA 16-I; whereas, six studies focused on differently decorated RADA 16-I; both a single and a dual functionalization were considered.

### 3.2 SAPs for sciatic nerve injury repair: focus on pre-clinical studies

Following a PNI, both the severity and the injury site can influence recovery. The severity of the injury depends on the number and size of the affected nerve bundles (e.g., partial vs complete nerve lesion). Not all nerves have the same capacity for recovery, and the type and location of the injury (e.g., proximal *versus* distal nerve, mixed nerve, motor/sensitive nerve) are crucial factors in predicting regeneration (Modrak et al., 2020).

The rat is the most used species in studies of peripheral nerve regeneration because, together with easy accessibility, its nerve fibers are comparable in size, fascicular organization, and morphology to human nerve fibers, making it a suitable sub-primate model (Geuna, 2015; Gordon and Borschel, 2017; DeLeonibus et al., 2021; Stocco et al., 2023a; Zhou et al., 2025). Comparing different strains (e.g., Sprague-Dawley, Buffalo, Brown Norway, Wistar-Furth, Wistar-Kyoto, Lewis), Lewis rats typically do not demonstrate self-mutilating behaviour (toe autotomy), unlike Sprague-Dawley rats (Carr et al., 1992); also Wistar-Kyoto are less prone to autotomy if kept in specific temperature conditions (Wiesenfeld and Hallin, 1981). However, studies involving the use of tubular devices combined with SAPs to support nerve regeneration have predominantly used the Sprague-Dawley strain (Wang et al., 2014; Wu et al., 2017; Lu et al., 2018; Lu et al., 2019; Yang et al., 2020; Yang et al., 2021a; Shen et al., 2022; Nune et al., 2017; Stocco et al., 2023b). While gaps of 10 mm (Wang et al., 2014; Lu et al., 2018; Lu et al., 2019; Yang et al., 2020; Shen et al., 2022; Nune et al., 2017; Stocco et al., 2023b) are most commonly studied, experiments with both larger [15 mm (Yang et al., 2021a)] and smaller [5 mm (Wu et al., 2017; Stocco et al., 2023b)] gaps have also been reported. Specifically, SAPs have been used for PNI recovery both as luminal fillers within nerve guidance conduits (Zhan et al., 2013; Wang et al., 2014; Wu et al., 2017; Lu et al., 2018; Lu et al., 2019; Yang et al., 2020; Yang et al., 2021; Shen et al., 2022) and as components integrated into the conduit walls (Nune et al., 2017; Fang et al., 2019; Stocco et al., 2023b). Here, SAPs as luminal fillers are focused; intriguingly, peptides can be injected in correspondence of the lesion where, sensing the environment, they organize (self-assemble) into a specific 3D structure, depending on sequence intrinsic/chemical characteristics. Specific conformations of the hydrogel can also be obtained exerting a control over pH, light, temperature, and enzymes (Zhang et al., 2021).

### 3.3 SAPs reported as luminal filler

In the wide panorama of SAPs, the only one that has been extensively applied for peripheral nerve regeneration in the form of luminal filler is RADARADARADARADA (RADA 16-I). Specifically, together with a reported experience considering an in-filled artery-derived conduit (Zhan et al., 2013), also guides based on chitosan (Lu et al., 2018; Lu et al., 2019; Yang et al., 2020; Yang et al., 2021a; Shen et al., 2022), poly (lactic-co-glycolic acid) (PLGA) (Wang et al., 2014), and Poly (L-lactic acid) (PLLA) (Wu et al., 2017) were improved by the addition within the lumen of RADA 16-I, eventually enriched with functional motifs (Supplementary Table 1).

RADA 16-I is a synthetic peptide made up of 16 amino acids and consisting of repeating units that are arginine (R), alanine (A) and aspartic acid (D). While R attracts water, A and D repel water. Depending on the pH level, RADA 16-I self-assemble into fibers; briefly, under acidic pH conditions, nanofibrils are observed; whereas, increasing pH or adding salt, higher order nanofibril scaffolds are obtained (Arosio et al., 2012). These consist of highly ordered β-sheet structures that are stabilized through non-covalent interactions (Santhini et al., 2022). Interestingly, RADA 16-I can self-assemble thus forming nanofibers even in the presence of physiological solutions/body fluids, enhancing its potential for applications in tissue engineering and regenerative medicine (Zhang et al., 2005). This 3D organization offers advantages in topography and porosity, closely mimicking the native ECM and providing a scaffold that supports regeneration (Wang et al., 2014). Additionally, RADA 16-I possesses intrinsic properties that promote differential cell growth, proliferation, and migration, making it a valuable material for influencing wound healing processes (Dzierżyńska et al., 2023).

#### 3.3.1 Results of RADA 16-I in sciatic nerve models

The initial studies on sciatic nerve regeneration through pure RADA 16-I hydrogels were reported by Zhan et al. (2013) and Wang et al. (2014).

Among autologous conduits, veins are a valuable option for nerve tubulization due to their wide availability and lower risk of harvesting-related complications compared to nerve grafts (Shintani et al., 2018). However, their tendency to collapse, and valve obstruction limit their use in large nerve defects (Rebowe et al., 2018; Rinker and Liau, 2011). To address these issues, Zhan et al. (2013) employed rat abdominal aorta segment filled with RADA 16-I nanofibers to bridge a 10 mm sciatic nerve gap. Two weeks after surgery, numerous NF200-positive axons and migrating SCs, that are critical for axons guidance (Chen et al., 2019), were observed at the proximal stump. Myelin Basic Protein (MBP) was absent in both RADA-filled and empty conduits but detectable proximally in the RADA group, suggesting early myelination; demyelination was more evident in controls. These results were consistent with those of Otake et al. (2020), who found region-specific myelination in collagen-filled conduits over 2–4 weeks. Zhan et al. (2013) also noted a nearly absent infiltration of macrophages (ED1^+^ elements) and lymphocytes (CD3^+^ elements) in the RADA group, suggesting a less inflammatory microenvironment. Considering that the macrophages play a crucial role in clearing damaged myelin sheaths debris, axon fragments, dead cells and foreign bodies (Wu et al., 2025), their lower presence in the RADA group may be associated with a more efficient regeneration. At 16 weeks, motor axon regrowth was confirmed *via* retrograde labeling, with significantly more labeled motoneurons in the RADA-filled group than in controls (1,041.5 ± 186.1 vs 470.2 ± 73.2) (Zhan et al., 2013).

Later, also Wang et al. (2014) investigated the regenerative effects of RADA 16-I as a luminal filler in PLGA conduits. At 16 weeks from surgery, it was observed NF200-positive axons aligned from proximal to distal ends and enhanced angiogenesis, known to facilitate SC migration (Fornasari et al., 2022; Cattin et al., 2015). Conversely, for empty conduits were confirmed the less encouraging regenerative outcomes that were previously highlighted by Zhan et al., 2013. Specifically, moderate axons were only detected in the proximal part of the device whereas the middle and the distal parts were sparsely populated (Wang et al., 2014). MBP immunostaining and TEM confirmed remyelination in the RADA group, even though axons and myelin sheaths were smaller than in naïve nerves (p < 0.05). Nevertheless, remyelination was significantly improved *versus* empty conduits (p < 0.05). It is well established that SCs have multiple roles following PNI: in addition to clearing debris and controlling inflammation, they are involved in axon growth and remyelination (Yang et al., 2021a). Experimental evidence may suggest that RADA 16-I creates a favorable environment for SCs within the conduit, as emphasized by comparison with the empty conduit data. The *g*-ratio (axon diameter/myelinated fiber diameter) is a key indicator of the maturity and functional health of regenerated myelinated nerve fibers (de Campos et al., 2014; Stocco et al., 2023b), considering this parameter, no statistically significant difference was observed within the cohort (Wang et al., 2014). Wang et al. (2014) also examined motoneuron survival and regeneration: about 86% of neurons survived in both syngeneic and RADA-filled conduits, compared to 74.2% in empty conduits; the same trend was observed also by retrograde labelling, with 57.1% ± 8.6% of labelled motoneurons in syngeneic grafts, 33.8% ± 7.3% in RADA-filled conduits and 19.1% ± 6.1% in empty conduits (p < 0.05).

Overall, the studies by Zhan et al. (2013) and Wang et al. (2014) highlight the role of unmodified RADA 16-I hydrogels in supporting peripheral nerve regeneration (gap: 10 mm) when used as a luminal filler of natural/synthetic conduits. Specifically, RADA 16-I supported axonal regrowth, early myelination, SCs migration, reduced inflammation, and improved motor neuron survival. Interestingly, compared to empty conduits, RADA 16-I was associated with significantly improved outcomes in structural/functional nerve repair, thus confirming its intrinsic bioactivity and adequacy for nerve tissue engineering.

#### 3.3.2 Results of RADA 16-I in muscle regeneration

Muscle dysfunction and atrophy with loss of muscle mass are typical features following PNI. Typically, four stages characterize muscle atrophy, namely,: oxidative stress, inflammation, mitophagy, and atrophic fibrosis. Additionally, since the musculoskeletal system includes both muscles and bones, damage to skeletal muscle can also involve bones with consequent functional and structural abnormalities (Yadav and Dabur, 2024). Following PNI, myoatrophy degree and target muscle recovery are effective indicators of nerve regeneration quality (Wang et al., 2023; Zhao et al., 2023). Typically, muscle reinnervation, motor recovery can be evaluated through morphological, histological, electrophysiological and gait evaluations.

Zhan et al. (2013) demonstrated that the incorporation of RADA 16-I into aorta-derived nerve conduits led to better muscle preservation compared to controls. At 16 weeks post-surgery, rats treated with RADA 16-I-filled grafts showed more regular and densely packed gastrocnemius myofibers, closely resembling healthy tissue. In contrast, the empty-conduit group exhibited atrophic myofibers and interstitial steatosis, typical of chronic denervation.

Further evidence supporting the use of nerve conduits in muscle recovery comes from Wang et al. (2014), who compared a syngeneic nerve graft, a RADA 16-I-filled PLGA conduit, an empty PLGA conduit, and a non-graft control (gap: 10 mm). The syngeneic group showed the best outcomes, with a myofiber transverse area ratio (injured/intact side) of 79.8% ± 8.5%, followed by the RADA 16-I group (39.9% ± 5.5%), the empty conduit group (26.9% ± 3.4%), and the non-graft group (18.1% ± 3.1%). A significant difference (p < 0.05) was calculated between these groups. Regarding muscle wet weight [injured/intact side wet weight ratio], it increased according to the following order: non-graft [28.38% ± 9.68%] > empty PLGA conduit [28.97% ± 11.98%] > RADA 16-I filled PLGA conduit [53.16% ± 9.18%] > syngeneic implant [62.45% ± 9.10%]; except for the empty conduit group *versus* non-graft group, the differences were statistically significant (p < 0.05).

Neuromuscular junctions (NMJs), which connect motor neurons to muscle fibers, has a fundamental role in muscle contraction and function (Iyer et al., 2021; Li et al., 2023; Sirago et al., 2023). Changes in NMJ size and morphology are closely associated with muscle type and performance (Mech et al., 2020). Wang et al. (2014) considered the NMJ size in longitudinal gastrocnemius muscle sections. In general, a reduction in NMJ size was observed in the operated limb compared to the naïve group (p < 0.05). NMJ dimensions improved across groups in the following order: non-graft < empty conduit < RADA 16-I-filled conduit < syngeneic graft, the latter showing no significant difference from the RADA 16-I group, indicating effective preservation of NMJ structure.

Motor deficits distinguish as important clinical signs in several neuromuscular diseases (Varejão et al., 2002); the foot and ankle have both a critical role in locomotion thus, gait evaluation through the Sciatic Functional Index (SFI) provides a useful measure of nerve function recovery (Varejão et al., 2002). Wang et al. (2014) observed an impaired foot positioning in nearly 90% of footprints from the non-graft control group; the 79.3% were “heel supported”, whereas only 10.5% were “palm supported.” In contrast, palm-supported steps were more frequent in the conduit groups, following this order: empty PLGA conduit > RADA 16-I-filled conduit > syngeneic graft, with statistically significant differences among all groups (p < 0.05).

Overall, studies by Zhan et al. (2013) and Wang et al. (2014) show that RADA 16-I-filled nerve conduits significantly improve muscle preservation, NMJ integrity, and functional recovery after PNI. Compared to empty or non-grafted controls, RADA 16-I treatment resulted in larger muscle fiber areas, greater muscle wet weight, and better-maintained NMJ morphology, closely matching outcomes seen with syngeneic grafts. Additionally, animals treated with RADA 16-I exhibited improved gait and motor function. These results highlight RADA 16-I’s effectiveness in supporting both nerve regeneration and muscle recovery.

### 3.4 Decorated RADA 16-I for nerve regeneration

Self-assembling peptides are versatile sequences that can bind small bioactive molecules favoring tissue regeneration (Hernandez et al., 2023). RADA 16-I suffers from a critical issue as its solutions are acid (pH 3–4) prior to hydrogel formation; consequently, cytotoxicity and inflammation may derive from this (Wu et al., 2017). To overcome these issues, considerable efforts have been dedicated to RADA 16-I modification by incorporating bioactive peptide sequences. These sequences both mitigate the negative effects of acidity and offer a promising alternative to traditional growth factors, suffering from rapid degradation, potential side effects, and high costs (Lu et al., 2019). Interestingly, RADA 16-I can be improved through covalent binding with various bioactive peptides without significantly compromising its gelation properties (Dzierżyńska et al., 2023). Considering the growth factors with intriguing effects on nerve regeneration, RADA 16-I has been functionalized with sequences from nerve growth factor (NGF) (Lu et al., 2018), vascular endothelial growth factor (VEGF) and BDNF (Lu et al., 2019). NGF has an important role in neurogenesis and neuroprotection, thus counteracting PNI. In the first 5–7 days after injury, SCs are involved in myelin debris degradation and this phase is fundamental to promote regeneration. According to Li et al. (2020), NGF accelerates this process. Additionally, NGF can stimulate neuronal survival, promote axon growth and their elongation (Li et al., 2020). VEGF plays a pivotal role in regulating vascular development and angiogenesis, which are fundamental to assure tissue repair. It also promotes neuron survival, neurite outgrowth, and SCs proliferation. The restoration of microvascular circulation at the injury site is crucial for determining the potential for sensory recovery (Höke et al., 2001). In line with this, PNI has been associated with an upregulation of VEGF mRNA expression (Nishida et al., 2018). The BDNF promotes the expression of molecules involved in axonal regeneration regulation (Sanna et al., 2017); also BDNF mRNA levels result to be increased in injured sciatic nerves since day 3 from nerve lesion and this upregulation lasts for several weeks suggesting the factor involvement in regeneration (Zhang et al., 2000; McGregor and English, 2019).

#### 3.4.1 Results of decorated RADA 16-I in sciatic nerve models

Most authors investigating the potential role of RADA 16-I as a luminal filler have explored the enrichment of the SAPs with various bioactive factor-derived motifs, either alone or in combination, to assess the possibility of synergistic effects in supporting sciatic nerve regeneration after injury. After nerve injury, administration of exogenous neurotrophic factors can mimic the effect of target organ-derived trophic factors release on neuronal cells (Terenghi, 1999).

One of the earliest and most representative examples is provided by Lu et al. (2018), who modified RADA 16-I by conjugating the NGF-derived peptide CTDIKGKCTGACDGKQC (CTD) and the BDNF-derived peptide RGIDKRHWNSQ (RGI) to the C-terminus of RADA 16-I, creating RADA/CTD, RADA/RGI and RADA/CTD + RGI variants. NGF and BDNF are known to support sensory and motor recovery (Sandoval-Castellanos et al., 2020). Regarding the optimal amount of functional motif to use, Horii et al. (2007) demonstrated that even a 1% bioactive motif combined with 99% RADA 16-I in a mixed scaffold can promote cell adhesion and proliferation. However, a content of 40%–70% was recognized as the most effective range. Accordingly, Lu et al. (2018) used a 50% functionalized peptide. *In vitro* analyses demonstrated that combining both sequences into a single construct (RADA/CTD + RGI) significantly improved SC viability and neurotrophic support compared to RADA alone or singly modified SAPs. This dual-functionalized hydrogel was subsequently evaluated *in vivo* using a 10 mm sciatic nerve gap in rats, where it outperformed hollow chitosan conduits and those filled with single-sequence SAPs in promoting axon regeneration. By 2 weeks post-surgery, animals implanted with RADA/CTD + RGI exhibited longer axonal extensions and more rapid regenerative growth, likely due to the creation of a 3D microenvironment that mimics the ECM and provides both structural guidance and molecular signals. At 12 weeks, RADA/CTD + RGI continued to show superior outcomes in terms of nerve fiber density and remyelination, closely resembling those observed in autografts, which were excluded from the direct comparison due to their different cellular environments (Fornasari et al., 2022).

Lu et al. (2019) investigated the regenerative effects of RADA16-I peptide hydrogels functionalized with VEGF- and BDNF-mimetic sequences (KLT and RGI, respectively) in both *in vitro* and *in vivo* models of PNI. Specifically, three variants were tested: RADA/KLT (50%/50%), RADA/RGI (50%/50%), and RADA/KLT + RGI (50%/25% + 25%). In accordance with previous findings (Lu et al., 2018), the dual-functionalized RADA/KLT + RGI scaffold promoted enhanced SC elongation with significant upregulation of neurotrophic and myelination-associated genes (NGF, BDNF, NRP2, PMP22, and S100) expression. Additionally, the expression of NCAM (a marker typically downregulated during myelination) (Wood et al., 1990), was lowest in the RADA/KLT + RGI group, suggesting a transition of SCs toward a myelinating phenotype.

*In vivo* experiments using a rat sciatic nerve injury model (Sprague Dawley) demonstrated that RADA/KLT + RGI hydrogels supported robust angiogenesis and axonal regeneration. By 6 weeks post-surgery, vascularization was highest in the RADA/KLT + RGI and RADA/KLT groups (comparable), followed by RADA/RGI and the autograft, with the hollow conduit showing the least vascular support. Given that an adequate and continuous oxygen supply is essential for tissue maintenance and nerve regeneration (Thibodeau et al., 2022), the observed angiogenesis likely contributed to the improved regenerative outcomes. At both 6 and 12 weeks, axonal regeneration (assessed by myelinated axon density, axonal diameter, and myelin sheath thickness) was comparable to that achieved with autografts. These findings highlight the therapeutic potential of the RADA/KLT + RGI scaffold as an effective alternative for long-gap peripheral nerve repair.

Being one of the most important cell-specific laminin motifs, IKVAV (Ile-Lys-Val-Ala-Val) peptide has an important role in cell–ECM interactions, signaling, and neurite outgrowth (Yergeshov et al., 2024). Additionally, as it can boost viability/maturation of neurons, it may have a role as a part of neuronal growth-stimulating devices (Farrukh et al., 2017) with a favorable effect over induced pluripotent stem cells-neural progenitor cells (Lam et al., 2015), and human neural stem cells (Li et al., 2014). The motif RGD (Arg-Gly-Asp) corresponds to a fibronectin-derived cell adhesion ligand. Presence of oligopeptides showing IKVAV or RGD epitopes, immobilized onto a substrate, can promote the adhesion, differentiation, or neurite outgrowth of neural progenitor cells/stem cells, embryonic neurons, and mesenchymal stem cells (Sun et al., 2016). In consideration of this, Wu et al., 2017 compared the performance of RADA 16-I with that of a mix made of RADA 16-I combined with the sequence RGD (RADA/RGD) and the sequence IKVAV (RADA/IKVAV) (from here on, RADA-Mix). A control group using PLLA conduits filled with saline was also included. Following *in vivo* implantation in a 5 mm sciatic nerve gap in rats, the RADA16-Mix group (RADA 16-I functionalized with RGD and IKVAV) demonstrated superior regenerative outcomes compared to RADA 16-I alone and saline controls. At 4 weeks, RADA16-Mix supported abundant, aligned axon growth, while the other groups showed disorganized or peripheral axonal distribution, with fissures and scar tissue present. By 8 weeks, RADA16-Mix maintained a uniform and parallel axonal orientation, unlike the random or radial growth seen in the RADA 16-I and saline groups. At 12 weeks, the RADA16-Mix graft continued to promote structured axon alignment and regeneration, outperforming the others in axon density and extension beyond the conduit. These findings confirm the enhanced capacity of RADA16-Mix to support long-term nerve repair and functional recovery.

A further example of dual-functionalized hydrogel was provided by Yang et al. (2020) who targeted a 10 mm sciatic nerve gap in rats. Specifically, chitosan conduits were filled with RADA16-I alone, RADA modified with RGI, and dual-functionalized RADA (RADA/IKVAV-GG-RGI and RADA/IKVAV/RGI). *In vitro*, the dual-functionalized hydrogels significantly enhanced SCs adhesion, elongation, and proliferation, as well as the expression and secretion of neurotrophic factors (NGF, BDNF, CNTF) and myelin-related genes (PMP22, MBP, NRP2). Moreover, the cells acquired an elongated morphology (suggesting myelination); conversely, they showed a roundish appearance on the RADA alone. One week after implantation, dual-functionalized hydrogels showed high expression of regeneration-related genes, with P0 levels in the RADA/IKVAV/RGI group comparable to that of the autograft. By 12 weeks, histological and TEM analyses confirmed that these scaffolds promoted higher myelinated fiber densities, larger axonal diameters, thicker myelin sheaths, and near-optimal *g*-ratios, with outcomes second only to the autograft and significantly superior to single-motif or unmodified RADA. IKVAV and RGI motifs, together, can enhance early nerve regeneration and promote robust myelination.

The importance of effective integration between biochemical and topographical cues was also demonstrated by Yang et al. (2022) who developed a composite system which combined aligned fibrin nanofiber hydrogel (AFG) with functionalized SAPs (fSAP: 25% RADA-IKVAV + 25% RADA-RGI + 50% RADA) to bridge a 15-mm sciatic nerve gap in rats. At 12 weeks from surgery, the AFG/fSAP group showed a significantly higher density of myelinated nerve fibers compared to AFG alone and hollow conduit controls, with values similar to that of autografts (12,194.2 ± 644.9 nerves/mm^2^). Additionally, the AFG/fSAP group showed significantly larger myelinated fiber diameters (5.30 ± 0.25 μm) and thicker myelin sheaths (0.73 ± 0.03 μm) than both the hollow and AFG groups (p < 0.01), indicating enhanced remyelination. The *g*-ratio values, ranging between 0.6 and 0.7, were most favorable in the AFG/fSAP group (0.67 ± 0.03), correlating with a greater number of large-diameter myelinated fibers. Molecular analyses performed 1 week after surgery revealed that regeneration-related genes (VEGF, BDNF, GDNF, CNTF, NGF) were significantly upregulated in the AFG/fSAP group compared to controls, suggesting a positive effect over angiogenesis, neuritogenesis, and myelination. Enhanced neuronal survival and axon growth (suggested by activation of the PI3K/Akt and MAPK pathways) with lower inflammation (suggested by reduced p38 and JNK phosphorylation) were also recognized (Huang et al., 2017). Overall, the study demonstrated that combining aligned fibrin nanofibers with functionalized peptides provides synergistic structural and biochemical cues that effectively accelerate nerve regeneration, with great potential in long-gap PNI repair.

Finally, Shen et al. (2022) explored the combined use of VEGF- and ECM-related peptides by preparing RADA/KLT, RADA/IKVAV, and RADA/KLT/IKVAV hydrogels. These constructs were evaluated in a 10 mm sciatic nerve gap model in rats. At 6 weeks from surgery, RADA/KLT and RADA/KLT/IKVAV groups were associated with the highest neovascular density (38.1 ± 3.6 and 38.5 ± 3.6, respectively), significantly higher (p < 0.01) than that displayed by the Hollow group (19.2 ± 3.1), RADA/IKVAV group (10.0 ± 2.1), and Autograft (7.4 ± 1.6) group. Quantitative analysis also confirmed significantly increased neovascular area ratios in these groups. Regarding remyelination, toluidine blue staining revealed that RADA/KLT/IKVAV and Autograft groups formed thick myelin sheaths and numerous myelinated axons, whereas the Hollow group had thin sheaths and fewer axons. The density of myelinated axons was significantly higher in the RADA/IKVAV and RADA/KLT/IKVAV groups compared to the Hollow and RADA/KLT groups, though still lower than the Autograft.

Several studies demonstrate that the functionalization of RADA 16-I hydrogels with bioactive motifs derived from neurotrophic factors (e.g., NGF, BDNF), angiogenic peptides (e.g., VEGF-mimetic KLT), and ECM-related sequences (e.g., IKVAV, RGD, RGI) significantly enhances peripheral nerve regeneration *versus* unmodified RADA or empty conduits. Furtherly, dual-functionalized hydrogels, with these motifs combined synergistically, improve Schwann cell activity, axonal growth, remyelination, angiogenesis, and motor recovery in animal models of sciatic nerve injury. Additionally, incorporating aligned nanofiber structures with functionalized SAPs boosts regeneration by providing both directional guidance and molecular cues. These biomimetic scaffolds can lead to outcomes approaching that of the autograft, thus offering promising alternatives for repairing long-gap nerve injuries.

#### 3.4.2 Results of decorated RADA 16-I in muscle regeneration

Across several studies, functional recovery of the sciatic nerve was assessed using different approaches, with multiple functionalized hydrogels showing outcomes comparable to autografts. The compound muscle action potential (CMAP) is the primary signal measured in motor nerve conduction studies; it reflects the summed electrical response of a muscle following stimulation of its motor nerve (Barkhaus et al., 2024). In Lu et al. (2018), the RADA/CTD + RGI group CMAP amplitudes were similar to the autograft group and significantly higher than RADA/CTD or RADA/RGI groups. Gait analysis at 4 weeks and gastrocnemius recovery at 6 weeks confirmed these results. Ultrasound imaging showed muscle elasticity ranked as: Autograft > RADA/CTD + RGI > RADA/RGI > RADA/CTD. Masson’s trichrome staining showed low collagen content in both the autograft and RADA/CTD + RGI groups. Cross-sectional muscle area was also similar between these two groups. In Lu et al. (2019), at 6 weeks post-surgery, the RADA/KLT + RGI and Autograft groups showed less gastrocnemius muscle atrophy than other groups, with muscle wet weight ratios of 34.5% ± 6.7% and 38.0% ± 6.0%, respectively. By 12 weeks, the RADA/KLT + RGI group had significantly greater muscle weight and fiber area (1,045.5 ± 310.5 μm^2^) than Hollow, RADA/KLT, and RADA/RGI, and was comparable to Autograft (1,138.2 ± 202.7 μm^2^). CMAP amplitude ratio in RADA/KLT + RGI (77.0% ± 7.1%) was close to Autograft (85.6% ± 3.1%), with shorter latency than other groups. CatWalk gait analysis showed improved SFI scores from 8 weeks onward, with RADA/KLT + RGI matching Autograft performance. Overall, RADA/KLT + RGI effectively reduced muscle atrophy and promoted functional recovery. Wu et al. (2017) observed increased NMJ reinnervation over time, with >60% reinnervation by 8 weeks in both RADA16-Mix and RADA16-I groups. At 12 weeks, although the RADA16-I group showed better outcomes, statistical differences were not significant. Gastrocnemius weight ratios improved over time, and gait-stance duration analysis highlighted RADA16-Mix as the best performing group. In Yang et al. (2020), at 12 weeks, dual-functionalized peptide hydrogels (IKVAV and RGI) enhanced CMAP amplitude and muscle reinnervation compared to Hollow, with SFI scores and muscle weight confirming motor recovery.

In Yang et al. (2021a), the AFG/fSAP group showed a CMAP amplitude of 11.86 ± 0.56 mV and latency of 1.64 ± 0.17 m, significantly better than Hollow and AFG, and close to autograft levels. Muscle weight ratios and fiber areas were also similar to autograft, and SFI values indicated superior motor recovery.

In Shen et al. (2022), the RADA/KLT/IKVAV group was associated with a functional and electrophysiological recovery 12 weeks post-surgery. CMAP peak amplitude was 78.53% ± 3.94%, close to Autograft (85.59% ± 3.15%) and significantly higher than that recorded for Hollow, RADA/KLT, and RADA/IKVAV groups. Also CMAP latency (1.29 ± 0.17 m), muscle elasticity at 6 weeks (61.53 ± 83.13) and muscle wet weight ratio at 12 weeks (75.87% ± 3.26%) were all comparable to that of Autograft. Regarding muscle fiber area, the measured values (1,078.70 ± 355.07 μm^2^) were similar to Autograft and significantly higher than that reported for other groups of the cohort. Gait analysis showed improved SFI (−56.06 ± 1.94) and stand/swing ratio (2.46 ± 0.43), again nearing Autograft outcomes. Overall, RADA/KLT/IKVAV hydrogel effectively promoted nerve regeneration and motor recovery, with performances that can be compared with that of autografts.

Multiple studies demonstrate that dual- or multi-functionalized RADA 16-I hydrogels significantly promote functional recovery of the sciatic nerve after injury, with outcomes comparable to autografts. CMAP amplitude, muscle weight and fiber area, NMJ reinnervation, and gait analysis, consistently show enhanced motor recovery in groups treated with conduits + functionalized SAPs. In particular, functionalization was based on different combinations of neurotrophic (e.g., NGF-, BDNF-derived), angiogenic (KLT), and ECM-mimetic (IKVAV, RGD, RGI) peptides. These complex hydrogels showed to reduce muscle atrophy, improve electrophysiological signals, and restore muscle elasticity and motor function, supporting their promise as effective synthetic alternatives to autografts for peripheral nerve repair.

## 4 Conclusion

Extracellular matrix shows a complex organization, characterized by a unique mesh of proteins and glycosaminoglycans that have both structural and functional roles. In fact, this arrangement not only provides mechanical support but also regulates biochemical signaling pathways that are fundamental for the ECM’s role in supporting cellular functions and thus maintaining tissue integrity. Considering this complexity, the design of biomimetic materials for peripheral nerve regeneration must account for specific features to be effective.

In recent years, SAP-based hydrogels have emerged as promising candidates for nerve repair, because of their ability to mimic the ECM’s multifunctional architecture. Overall, results from the reviewed studies highlight that SAPs presence within nerve conduits has a positive impact on nerve continuity and function restoration when compared to empty devices. Moreover, combining RADA 16-I (the only reported as luminal filler for sciatic nerve repair, to our knowledge) with multiple bioactive sequences was shown to boost conduits regenerative potential with positive morphometric and functional outcomes. However, the interpretation of these results requires careful consideration of experimental variables, in particular the nerve gap length addressed.

Each animal species has a specific threshold (i.e., the critical-sized nerve gap), beyond which spontaneous peripheral nerve regeneration and target reinnervation are rarely observed. In rats, this critical gap is approximately 15 mm, which corresponds to around 40 mm in humans (Podsednik et al., 2022). Except for Yang et al. (2021a), who reported on a 15 mm sciatic nerve defect in Sprague-Dawley rats, most studies, employed conduits (filled with SAPs or SAP + conjugate) to target lesions ranging from 5 to 10 mm. However, it is well established that gaps of 10 mm or less may undergo spontaneous regeneration, possibly failing to adequately reflect the complexities of nerve repair (Lundborg et al., 1982; Casal et al., 2018). Therefore, future studies should consider longer gaps (15 mm or more), as in this case further design modifications, to support consistent and effective regeneration, are required.

Additionally, a further critical evaluation of the studies considered here reveals a certain variability in methods adopted to verify the filled-conduits associated outcomes, thus limiting their translational applicability. While several works, following morphometric analyses, report improvements in axons growth, early remyelination, Schwann cell migration, reduced inflammation, muscle preservation, and NMJ restoration (often with outcomes comparable to autografts in terms of CMAP amplitude, muscle fiber area, and gait recovery) these conclusions derive from the application of different protocols with different end-points, gap lengths, and outcome measures (Supplementary Table 1). Furthermore, direct comparisons to autografts were not always reported (Wang et al., 2016; Wu et al., 2017). To assess the clinical potential of RADA-based constructs in an accurate manner, future studies should adopt standardized approaches based on the combination of robust morpho-structural quantification methods (e.g., total axons number and density, axons diameter, fiber diameter, myelin thickness, *g*-ratio), functional assessments of both motor recovery (electrophysiology to determine nerve conduction velocity, and CMAP measuring nerve signal transmission and muscle response [Lu et al., 2018; Lu et al., 2019; Yang et al., 2020; Yang et al., 2021b; Shen et al., 2022)] and sensory recovery [evaluation of sensory threshold through different types of stimuli (Sonohata et al., 2023)], long-term follow-up, and direct autograft comparisons. Only through a whole and harmonized approach it may be possible to validate the regenerative potential of these promising biomaterials with also possible translation into clinical practice. Preclinical studies using strains other than Sprague-Dawley may be valuable to minimize the autotomy-related issues commonly observed with them.

Given the novelty of using SAPs as luminal fillers, it can be assumed that the current research represents an exploratory phase in this field. Such multifunctional 3D structures may offer tailored biochemical and structural cues with the ability to support tissue repair, thus advancing the development of next-generation biomaterials for nerve regeneration. As reported by Yang P. P. et al. (2021), due to the vast combinatorial space of even short peptides, many SAPs sequences can be developed and the identification of the most effective for a specific tissue target remains a significant challenge. Certainly, intense research efforts in this field may lead to fully successful outcomes in nerve regeneration, potentially achieving results superior to those obtained with autografts.
