# Supplementary material for: Self-assembling peptides for sciatic nerve regeneration: a review of conduit microenvironment modeling strategies in preclinical studies
Source: Front Cell Dev Biol. 2025 Aug 13;13:1637189. doi: 10.3389/fcell.2025.1637189 (PMC12380886; doi:10.3389/fcell.2025.1637189)
Supplement: Supplementary file 1 [file Table1.docx]

**Table 1. SAPs as luminal fillers: preclinical studies description**

| REFERENCE | NERVE CONDUIT MATERIAL | SAP  VOLUME | SAPS CHARACTERIZATION STUDIES | ANIMAL SPECIES | EXPERIMENTAL GROUPS | SCIATIC NERVE GAP LENGTH | END-POINT | ANALYSES | MAIN OUTCOMES |
| --- | --- | --- | --- | --- | --- | --- | --- | --- | --- |
| Zhan et al., 2013 | Blood vessel conduit | 1% SAPNS solution made of RADA16-I peptide  To fill the conduit full length | **---** | Female Sprague-Dawley rats (adult) | - Aorta conduit filled with RADA 16-I (n=18)  - Empty aorta conduit (n=13)  - Defect  without treatment (n=5) | 10 mm | - n=3 rats of conduit groups were sacrificed at 2w;  - 16 w after surgery | - **Behavioral testing**   - SFI (6,9,15 w after surgery)   - **Retrograde tracing and spinal motoneurons quantification** - **Sciatic nerve**   - Electrophysiology measurement  - Histomorphometry of axonal regeneration (NF200)  - CNPase and MBP for Schwann cells and matured myelin (double immunostaining)  - TEM  - fiber diameter and *g*-ratio  - neuroinflammation (double immunostaining: ED1 for macrophages and CD3 for lymphocytes)   - **Gastrocnemious muscles**   - Weight ratio of the injured side to the intact side  **-** H&E staining on longitudinal sections  - TEM | - Aorta conduit filled with RADA 16-I enabled the peripheral axons regeneration beyond the gap.  - RADA16-I enabled and enhanced motoneuron protection, axonal regeneration and  remyelination  - Target reinnervation and functional recovery induced by the SAPNS-based conduit |
| Wang et al., 2014 | PLGA porous membrane; thickness: 100 µm; inner Ø: 1.2 mm | RADA16-I peptide  To fill the conduit full length | --- | Female Sprague-Dawley rats (adult) | - Syngeneic peripheral nerve grafts (n=12)  - PLGA conduits+1% RADA16-I solution (n=12)  - Empty PLGA conduit (n=12)  - Non-graft control (n=12)  - Naïve control without injury (n=6) | 10 mm | 16 w after surgery | - **Behavioral testing**   - SFI (15 w after surgery)   - **Retrograde tracing** (15 w after surgery) - **Sciatic nerve**   - TEM and morphometric study (i. myelin thickness, ii. axonal diameter, iii. *g-*ratio of the myelinated nerve fiber [axon diameter/whole myelinated fiber diameter], iv. myelinated axon ratio [myelinated axons number/total axons number])  - Immunofluorescence (double, NF200/MBP)  - Alkaline phosphatase histochemistry (vascularization)   - **Gastrocnemious muscles**   - Weight ratio of the injured side to the intact side  - Histomorphometry (muscular fibers number on H&E-stained sections; neuromuscular junction area after staining with α-BTX)   - **5^th^ lumbar segment of the spinal cord**   - Surviving and regenerating motor neurons | - PLGA conduits+1% RADA16-I solution group had a larger number of growing and extending axons, a markedly increased Ø of regenerated axons and a greater thickness of the myelin sheath in the conduit.  - Increase in the size of the neuromuscular junction and myofiber diameter in the target muscle |
| Wu et al., 2017 | PLLA electrospun conduit | - RADA16Mix (RADA16-RGD + RADA16-IKVAV)  20 µL | --- | Female Sprague-Dawley rats (adult) | - RADA16-I (n=26)  - RADA16Mix (n=29)  - Saline group (n=18)  - Control group (n=15) | 5 mm | - 4 w post-surgery  - 8 w post-surgery  - 12 w post-surgery | - **Behavioral testing**   - Motor function analysis by gait-stance duration test   - **Sciatic nerve**   - Immunofluorescence (NF200)  - H&E  - Morphometric study (total axon number at proximal/midpoint/distal junction site)   - **Gastrocnemious muscle**   - Weight ratio of the injured side to the intact side  - Double immunostaining (α-BTX for motor endplate and anti-NF200 for neurofilament)  - Evaluation of reinnervation quantifying the overlapping neurofilament and α-BTX immunoreactivity  - Reinnervation rate: fully innervated motor endplates/total motor endplates | - RADA 16-Mix showed homogenous  structure after transplantation in the conduit  - RADA 16-I graft was  composed of the hierarchical overlapping dense bulk with long  and narrow cavities along the radial direction of the conduit  - In the RADA  16-Mix hydrogel graft, the axons grew in parallel rows along the  axial direction of the electrospun conduit  - In the RADA 16-I hydrogel graft, axons extended along the wall surfaces of  the cavities, seldom axons were found to grow into the dense hydrogel  bulk.  - More axons regenerated in the RADA 16-Mix graft than those regenerated in  RADA 16-I graft  - In the distal part of the transected nerve, RADA  16-Mix group failed to show significantly more regenerated axons versus the other two grafted groups, even with the control  - Analysis of reinnervated  endplates and behavior test showed that  RADA 16-Mix group failed to display any significant advantage over other groups: although RADA 16-Mix outperformed RADA 16-I and saline in supporting axonal regeneration, it  is still not sufficient to let axons grow to the distal part of transected nerve to re-establish neuro-muscular junction |
| Lu et al., 2018 | Chitosan | - RADA 16-I/CTD  - RADA/RGI  - RADA/CTD+RGI  *Not specified* | - Circular dichroism  - Atomic Force Microscopy  - Morphology by SEM  - Rheometry  - 3D culture study: rat Schwann-cells/scaffold interaction, 3 days (S100 and confocal laser scanning microscope)  - 2D culture study: dorsal root ganglia – scaffold interaction, over 6 days (anti-tubulin and confocal laser scanning microscope)  - Analysis of PC12 cell neurite outgrowth on hydrogels | Male Sprague-Dawley rats (adult) | - Hollow chitosan conduit (n=10)  - Chitosan+RAD/CTD nanofiber hydrogel (n=10)  - Chitosan+RAD/RGI nanofiber hydrogel (n=10)  - Chitosan+RAD/CTD+RGI nanofiber hydrogel (n=10)  - Autograft (n=10) | 10 mm | - 2 w post-surgery  - 6 w post-surgery  - 12 w post-surgery | - **Behavioral testing**   - Evaluation of motor functional recovery at 2, 4, 6, 8,10 and 12 w  - SFI   - **Sciatic nerve**   - Immunohistochemistry (NF200, regenerated axons; P0, myelin protein) – 2 w  - Toluidine blue, distal ends (myelinated axons density)  - TEM , distal ends (myelin sheaths thickness, myelinated nerve fibers diameter) - 12 w, distal ends   - **Electrophysiological analyses**   *(12 w after surgery)*   - **Gastrocnemious muscles**   - Ultrasonography to assess the morphology and the elasticity of the gastrocnemious – 6 w  - Wet weight ratio analysis – 12 w  - Masson’s trichrome staining, mean cross-sectional area of the fibers – 12 w | - *In vitro,* the hydrogels exhibited good cell compatibility.  - The self-assembling hydrogels became colonized with SCs and DRG, and can promote neurite outgrowth of PC12 cells.  *- In vivo*, the combination of CTD+RGI accelerated axonal regeneration with good functional recovery, in accordance with histological and morphological analyses and electrophysiological and behavioral evaluations |
| Lu et al., 2019 | Chitosan | - RADA  - RADA/KLT  - RADA/RGI  - RADA/KLT/RGI  To fill the conduit full length | - Circular dichroism  - Atomic Force Microscopy  - Morphology by SEM  - Rheometry  - Rat Schwann-cells adhesion and morphology  - Proliferation studies with HUVECs (1, 4 and 7 days)  - qRT-PCR, 7 days (NCAM, NGF, BDNF, NRP2, PMP22, S100. | Male Sprague-Dawley rats (adult) | - Hollow chitosan conduit (n=12)  - Chitosan+RAD/KLT (n=12)  - Chitosan+RAD/RGI (n=12)  - Chitosan+RAD/KLT/RGI (n=12)  - Autograft (n=12) | 10 mm | - 6 w after surgery  - 12 w after surgery | - **Behavioral testing**   - Evaluation of motor functional recovery at 2, 4, 6, 8,10 and 12 weeks  - SFI   - **Sciatic nerve**   - H&E, distal nerve segment (density of newly formed blood vessels) – 6 w  - Toluidine blue, middle nerve segment (myelinated axons density) – 6 and 12 w  - TEM, middle regenerated nerve (diameters of fibers and myelinated axons, thickness of myelinated sheath, *g*-ratio) – 12 w   - **Gastrocnemious muscles**   - Masson’s trichrome staining, muscle fiber area – 6 and 12 w  - Ratio between the wet weight of the gastrocnemious and muscle fiber area percentage from the injured and contralateral sides – 6 and 12 w   - **Electrophysiological analyses**   (at 12 w after implantation) | - *In vitro* analyses with cells showed that the functionalized peptide hydrogel scaffold effectively promoted the SCs pro-myelination, and the adhesion/proliferation of HUVECs compared with scaffolds presenting VEGF- or BDNF-mimetic peptide epitope alone  - *In vivo*, the functionalized peptide hydrogel significantly ↑ the number of newly formed blood vessels, regenerating axons density, the morphometric analysis of the regenerated muscles and the electrophysiological findings  - The two bioactive motifs have a synergistic effect of on peripheral nerve regeneration |
| Yang et al., 2020 | Chitosan | - RADA/IKVAV (50% RADA + 50% RADA-IKVAV)  - RADA/RGI  (50% RADA + 50% RADA-RGI)  - RADA/IKVAV-GG-RGI  (50% RADA + 50%RADA-IKVAV-GG-RGI)  - RADA/IKVAV/  RGI  (50% RADA + 25% RADA-IKVAV + 25% RADA-RGI)  *Not specified* | - Circular dichroism  - Atomic Force Microscopy  - Pore size by SEM  - Rheological properties  - Rat Schwann-cells adhesion  and morphology | Male Sprague-Dawley rats (adult) | - Hollow chitosan nerve conduit (n=8)  - Chitosan nerve conduit + RAD/IKVAV hydrogel (n=8)  - Chitosan nerve conduit + RAD/RGI hydrogel (n=8)  - Chitosan nerve conduit + RAD/IKVAV-GG-RGI hydrogel (n=8)  - Chitosan nerve conduit + RAD/IKVAV/RGI hydrogel (n=8)  - Autograft (n=8) | 10 mm | 12 w after surgery | - **Behavioral testing**   - Evaluation of motor functional recovery at 2, 4, 6, 8,10 and 12 w  - SFI   - **Relative gene expression at the lesion site**   - NGF, BDNF,CNTF, IGF-2, S100, MBP, NCAM, PMP22, NRP2, VEGF,P0   - **Sciatic nerve**   - Evaluation of myelinated axons density (Toluidine blue stained sections)  - Thickness of myelin sheaths, myelinated axon diameter; thickness of myelin sheath, diameter based *g*-ratio, perimeter based *g*-ratio (TEM)   - **Electrophysiological recovery** - **Gastrocnemious muscles**   - Muscle wet weight ratio  - Cross-sectional area of the muscle fibers (Masson’s Trichrome stained sections) | - Both IKV and RGI improved SCs adhesion on hydrogels, increased cell spreading and elongation.  - SCs cultured on hydrogels with IKV and RGI showed ↑ gene expression of NGF, BDNF, CNTF, PMP22 and NRP2, and ↓ gene expression of NCAM versus those cultured on other three groups (7-day incubation).  - ↑ secretion of NGF, BDNF, and CNTF by SCs on dual-functionalized peptide hydrogels (after 3 days).  - Neurotrophin and myelin-related genes expressions in the nerve grafts, in SAP and Autograft groups were higher than in Hollow group (1 week after implantation); S100 expression in groups containing both IKVAV and RGI was significantly higher than that in groups containing either IKVAV or RGI hydrogels  - Regenerated nerves morphometric parameters/electrophysiological performance and innervated muscle weight/ muscle fibers remodeling, and motor function showed that RAD/IKVAV/RGI and RAD/IKVAV-GG-RGI hydrogels could markedly improve axonal regeneration with enhanced re-myelination and motor functional recovery through the synergetic effect of IKV and RGI functional motifs. |
| Yang et al., 2021a | Chitosan | - 3D aligned fibrin nanofiber hydrogel+RGI (BDNF-mimetic peptide+IKVAV) | - Hydrogels water content  - FTIR spectra  - Microstructure by micro-CT imaging (fibre alignment)  - Morphology by SEM  - Fibrin fibers/SAP nanofibers interaction by TEM  - Rheological properties  - Atomic Force Microscopy for hydrogels stiffness evaluation  - Molecular docking between fibrinogen and SAPs  - Proton nuclear magnetic resonsnce to further verify molecular interaction of functionalized SAP and fibrin  - *In vivo* degradation (*in vivo* fluorescence images at 1,4,7,10 and 14 days; harvest at 7 and 14 days post-operatively)  - Schwann cell morphology and neurotrophin (CNTF, BDNF, NGF) secretion after 3 days, by ELISA | Male Sprague-Dawley rats (adult) | - Autograft group (n=11)  - Hollow group (empty chitosan tube) (n=11)  - Chitosan+ aligned fibrin nanofiber hydrogel (n=11)  - Chitosan+ aligned fibrin nanofiber hydrogel+RGI (n=11) | 15 mm | 12 w after surgery | - **Behavioral testing**   - Evaluation of motor functional recovery at 2, 4, 6, 8,10 and 12 w  - SFI   - **qRT-PCR**   - 1 week after surgery (NGF, BDNF, CNTF, GDNF, S100, NF200, NGFR, CD31, NRP2, VEGF, NRP1, PTN, LIF, Nestin; GAP43, LN, Gas6)   - Western blot analysis   - 1 week after surgery (phospho-AKT; AKT; phospho-ERK; ERK; phospho-p38; p38; JNK; phosphor-JNK; β-actin)   - **Sciatic nerve**   - Morphometric evaluation of axonal regeneration at the distal ends of the grafts: average of myelinated nerve fiber density on toluidine blue images; thickness of myelin sheaths, diameter of myelinated nerve, *g*-ratio on TEM images   - **Electrophysiological analyses** - **Gastrocnemious muscles**   - Muscle wet weight ratio  - Masson’s trichrome staining (cross-sectional area of the muscle fibers) | - The hydrogel shows an aligned structure, high water content, appropriate mechanical properties and suitable biodegradation  Profile  - The hydrogel ↑ the alignment and neurotrophin secretion of primary SCs in vitro  - Fibrin fibers/SAP hydrogel show satisfactory morphological/functional recovery in myelinated nerve fibers and  innervated muscles.  - The motor function recovery supported by fibrin fibers/SAP hydrogel is comparable with that  of autografts.  - Fibrin fibers/SAP hydrogel ↑ the regeneration-associated gene expression and activates the PI3K/Akt and MAPK signaling pathways in the regenerated nerve. |
| Shen et al., 2022 | Chitosan | - RADA16-I  - RADA/IKVAV  - RADA/KLT  - RADA/KLT/IKVAV | - Circular dichroism  - Atomic Force Microscopy  - Morphology by SEM  - Proliferation and differentiation of SCs (staining with P0 and S100; visualization by laser confocal scanning microscopy; rate of P0 and S100 double positive cells). Proliferative ability at 1, 3 and 5 days from seeding | Male Sprague-Dawley rats (adult) | - Autograft group (n=12)  - Hollow chitosan (n=12)  - Chitosan+RAD/KLT nanofiber hydrogel  - Chitosan+RAD/IKVAV nanofiber hydrogel  - Chitosan+RAD/KLT/IKVAV nanofiber hydrogel | 10 mm | - 6 w after surgery  - 12 w after surgery | - **Behavioral testing**   - Evaluation of motor functional recovery at 2, 4, 6, 8,10 and 12 w  - SFI   - **Sciatic nerve**   - Masson’s trichrome staining (neovascular count; neovascular area ratio per view field) – 6 w  - Toluidine blue O, myelinated nerve fibers densityat the distal ends – 12 w  - TEM, myelinated fibers density and myelin sheath thickness – 12 w   - **Electrophysiological evaluation** – 12 w - **Gastrocnemious muscles**   - Ultrasonography to assess the morphology and the elasticity of the gastrocnemious – 6 w  - Wet weight ratio analysis – 12 w  - Masson’s trichrome staining, mean cross-sectional area of the fibers – 12 w | - SCs could adhere to and proliferate on the surface of peptide gels showing good cell compatibility.  *-* The functional recovery in the RAD/KLT/IKVAV group and autologous graft group were significantly faster than in the hollow conduct group and the other two SAP gels group alone  - Histological analyses proved a increased axonal and SCs regeneration within the reconstructed nerve gap by combined therapy. |

BDNF, brain derived neurotrophic factor; CTD, CTDIKGKCTGACDGKQC peptide; H&E, Haematoxylin and Eosin; HUVECs, Human Umbilical Vein Endothelial Cells; MBP, myelin basic protein; NCAM, neuronal cellular adhesion molecules; NGF, nerve growth factor; NRP2, neuropilin-2; OxPVA, oxidized polyvinyl alcohol; PLGA, poly(lactic-co-glycolic acid); Ø, diameter; PLLA, Poly-L-(lactic acid); PMP22, peripheral myelin protein 22; PTN, pleiotophin; SAP, Self-Assembling peptide; SCs, Schwann cells; SFI, Sciatic Functional Index; TEM, Transmission Electron Microscopy; w, weeks; α-BTX, α-bungarotoxin; Ø, diameter; ↓, decreased; ↑, enhanced; %, percentage; CNPase, 2',3'-cyclic nucleotide-3'-phosphodiesterase
